# A Comparative Study of Biodegradation of Nickel and Chromium from Space Maintainers: An *in vitro* Study

**DOI:** 10.5005/jp-journals-10005-1280

**Published:** 2015-04-28

**Authors:** Ashish Anand, Arun Sharma, Piush Kumar, Meera Sandhu, Shobhit Sachdeva, Vinod Sachdev

**Affiliations:** Postgraduate Student, Department of Pedodontics and Preventive Dentistry, ITS Centre for Dental Studies and Research, Muradnagar, Uttar Pradesh, India; Professor and Head, Department of Pedodontics and Preventive Dentistry, Institute of Dental Studies and Technologies, Modinagar, Uttar Pradesh India; Associate Professor, Department of Orthodontics, ITS Centre for Dental Studies and Research, Muradnagar, Uttar Pradesh, India; Professor, Department of Pedodontics and Preventive Dentistry, ITS Centre for Dental Studies and Research, Muradnagar, Uttar Pradesh, India; Senior Lectuerer, Department of Pedodontics and Preventive Dentistry, ITS Centre for Dental Studies and Research, Muradnagar, Uttar Pradesh, India; Professor and Head, Department of Pedodontics and Preventive Dentistry, ITS Centre for Dental Studies and Research, Muradnagar, Uttar Pradesh, India

**Keywords:** Artificial saliva, Chromium, Nickel, Space maintainers.

## Abstract

**Objective:** The aim of the study was to compare and evaluate the *in vitro* biodegradation of nickel and chromium from space maintainers, made of three different companies, i.e (Dantaurum, Rocky mountain and Dtech) in artificial saliva.

**Materials and methods:** The study comprised of 30 space maintainers out of which 10 were fabricated using Dantaurum, 10 using Rocky mountain and 10 using Dtech band materials. Stainless steel wire (Dantaurum, Rocky mountain and Konark) was used for making loops and Leone solder and flux was used for soldering. Each group was further divided into four subgroups containing 1, 2, 3 and 4 space maintainers respectively. The space maintainers in each subgroup were placed in separate glass beakers containing 100 ml of artificial saliva at 37°C for 4 weeks. Salivary samples from each beaker was analyzed for nickel and chromium ions separately on days 1, 7, 14, 21 and 28 days using inductively coupled plasma-optical emission spectrophotometer.

**Results:** Total release of nickel and chromium from all band and loop space maintainers ranged from 0.020 to 1.524 ppm and 0.002 to 0.289 ppm respectively. The release of nickel and chromium between the groups and within the groups was not significant (p < 0.5).

**Conclusion:** There was no substantial release of nickel and chromium from space maintainers made of Dantaurum, Rocky mountain and Dtech which could cause any toxicity.

**How to cite this article:** Anand A, Sharma A, Kumar P, Sandhu M, Sachdeva S, Sachdev V. A Comparative Study of Biodegradation of Nickel and Chromium from Space Maintainers: An *in vitro* Study. Int J Clin Pediatr Dent 2015; 8(1):37-41.

## INTRODUCTION

Stainless steel crowns and space maintainers are widely used in pediatric dentistry. These are made-up of base metal alloys along with iron, nickel and chromium as the main constituents. Nickel is incorporated in all austenitic stainless steel alloys to stabilize the austenitic phase, improve the tarnish resistance of the alloy, and decrease the ductility, while chromium is added to facilitate the formation of anticorrosive passivating film on the alloy surface.^1^

The warm and moist oral aerobic condition in the mouth offers an aggressive environment for electrolytic and electrochemical activity, thus offering an ideal environment for the biodegradation of metals consequently facilitating the release of metals and their corrosion products.^2^

The release of metal ions, especially nickel from dental alloys, is a possible concern because of its local and systemic toxicity, immunogenic, mutagenic and chemostatic effects.^3^ There is a possibility of nickel and chromium released from space maintainers, stainless steel orthodontic bands, brackets and wires eliciting an adverse reaction.

The nickel content in glandular saliva is largely influenced by dietary intake.^4^ Potential health effects from exposure to nickel and chromium include hypersensi-tivity, allergic reaction and ulceration of oral mucosa. Nickel complexes in the form of arsenides and sulphides can be carcinogenic and mutative; even at nontoxic concentrations nickel might induce DNA alterations mainly through base damage and DNA- strand scission.^3^ Besides originating from orthodontic appliances, nickel may be accumulated from saliva and food.

The present study was carried out to evaluate *in vitro* biodegradation of space maintainers made of three different company band materials (Dantaurum, Rocky mountain and Dtech) using Inductively coupled plasma-optical emission spectrophotometer and to also evaluate the concentration of nickel and chromium ions present in salivary samples following 1, 7, 14, 21 and 28 days.

## MATERIALS AND METHODS

The present study was carried out in the Department of Pedodontics and Preventive Dentistry at ITS Centre for Dental Studies and Research and National Test house, Ghaziabad, Uttar Pradesh.

The study comprised of 30 space maintainers out of which 10 were fabricated using Dantaurum, 10 using Rocky mountain and 10 using Dtech band materials. Each group of 10 space maintainers were further divided into four subgroups. Subgroups 1, 2, 3 and 4 containing 1, 2, 3 and four space maintainers respectively.

### Methodology

Ideal casts were obtained simulating second primary molar. Band and loop space maintainers were fabricated on first permanent mandibular molar. Ten space main-tainers were fabricated using Dantaurum wire and bands, 10 were fabricated using Rocky mountain wire and band and 10 were fabricated using Dtech bands and Konark wire. Leon solder and flux were used for soldering. They were later trimmed and polished.

The space maintainers in each subgroup were placed in separate glass beakers containing 100 ml of artificial saliva at 37°C for 4 weeks. The required amount of salivary sample was drawn out from each beaker and analysed for nickel and chromium ions separately on days 1, 7, 14, 21, 28 days using inductively coupled plasma-optical emission spectrophotometer.

*Measurement techniques*: The analysis was performed by inductively coupled plasma-optical emission spectro-photometer (Model no. Optima DV 2100 make perkin element) which was based on the principle of emission technology. The minimum detection limit of this instrument was 0.1 parts per billion. It was based on the principle of emission technology. The test solution was nebulized by the nebuliser and sprayed in plasma chamber, where electron was emitted on the particular wavelength of the particular element in the presence of plasma (at very high temp, i.e 12000 K°). The elements in the solution were determined qualitatively and quantitatively from the wavelengths and intensities of the emissions from excited atoms and ions of the emissions spectra utilized for element determination, the emission spectra from atoms were classified as neutral lines, and the emission spectra from ions were classified as ionic lines.

*Procedure*: Two percent nitric acid was used as a calibration blank. The instrument makes the background zero then the calibration of instrument was performed. For calibration standards of elements which were detected in the samples were taken and accurate standards for calibration were obtained which has international tracebility. For calibration, nickel and chromium having concentration of 0.01, 0.5, 1.0, 5.0, 10.0 ppm or mg/1 was used. After using the standard of 0.01, 0.5, 1.0, 5.0, 10.0 ppm, nickel and chromium solution (prepared in 2% HNO_3 _blank) calibration graph was plotted which was in the form of a linear line and the correction factor should be 0.9899 to 0.9999. In the study undertaken, the correction factor of nickel and chromium was 0.9999.

After calibration of instrument, analysis of the samples for nickel and chromium was done where ultra-pure water was used, which was having highest purity. The samples reading were taken atleast three times, the average values of three readings was taken as a final value.

## RESULTS

Thirty space maintainers (band and loop) made of three different materials were used for the purpose of study. These space maintainers were placed in separate glass beakers containing 100 ml of artificial saliva at 37°C for 4 weeks and release of nickel and chromium from space maintainer were evaluated. Total release of nickel and chromium from all band and loop space maintainers ranged from 0.020 and 1.524 ppm (Tables 1 and 2) and 0.002 to 0.289 (Tables 3 and 4) ppm respectively. The release of nickel and chromium between the groups and within groups was not significant (p < 0.5) (Tables 3 and 4).

## DISCUSSION

Metallic alloys are routinely used in dentistry in a variety of applications like fabrication of orthodontic appliances, fabrication of prosthetic appliances and indirect restoration of teeth. Most metallic alloys have definite composition of their own; usually varying from one another with one thing common among all that is the occurrence of trace elements.^5^

There has been an increased interest among dental and biomedical professionals regarding the side effects associated with the use of metallic materials, specially biomaterials.

The general mechanism for the corrosion and subsequent release of metal ions from stainless steel involves loss of the passivating layer of chromium oxide and chromium hydroxide that forms on the surface upon contact with oxygen.^6,7^

Nickel is a strong sensitizer and one of the most common cause of contact allergies and has carcinogenic and mutagenic effects. Allergic response to nickel-containing alloys is mainly type 4 hypersensitivity reaction, i.e. cell mediated reaction by t-lymphocytes. It has been suggested that long-term exposure to nickel-containing dental materials may adversely affect both human monocytes and oral mucosal cells. In addition to nickel, chromium and cobalt ions also cause hypersensitivity and dermatitis. These metals can also induce cytotoxicity and genotoxicity.^4^

**Table 1:** **Table 1:** Release of nickel ions in subgroups 1 and 2 in ppm

				*Release of nickel ions in subgroup 1 in ppm*									*Release of nickel ions in subgroup 2 in ppm*								
*Days*				*Dantaurum*		*Rocky mountain*				*Dtech*				*Dantaurum*		*Rocky mountain*				*Dtech*	
1				Nil		0.026				Nil				Nil		0.020				Nil	
7				Nil		0.044				Nil				Nil		0.037				Nil	
14				Nil		Nil				Nil				Nil		0.028				Nil	
21				Nil		Nil				Nil				Nil		Nil				Nil	
28				Nil		Nil				Nil				Nil		Nil				Nil	
Total release (ppm)				Nil		0.070				Nil				Nil		0.085				Nil	
		*Analysis of variance for nickel in subgroup 1*										*Analysis of variance for nickel in subgroup 2*									
*Source of variance*		*Sum of squares*		*Degree of freedom (DF)*		*Mean square*		*Variance ratio (F)*		*p-value*		*Sum of squares*		*Degree of (DF) freedom*		*Mean square*		*Varian ratio(F)*		*p-value*	
Between materials		0.001		2		0.000		2.402		0.133		0.001		2		0.000		5.217		0.023	
Within days		0.002		12		0.000						0.001		12		0.000					
Total		0.002		14								0.002		14							

**Table 2:** **Table 2:** Release of nickel ions in subgroups 3 and 4 in ppm

				*Release of nickel ions in subgroup 3 in ppm*									*Release of nickel ions in subgroup 4 in ppm*								
*Days*				*Dantaurum*		*Rocky mountain*				*Dtech*				*Dantaurum*		*Rocky mountain*				*Dtech*	
1				0.139		0.112				0.012				1.177		0.107				Nil	
7				0.227		0.153				Nil				1.524		0.129				Nil	
14				0.233		0.177				Nil				1.157		0.04				0.009	
21				Nil		Nil				Nil				Nil		Nil				Nil	
28				Nil		Nil				Nil				Nil		Nil				Nil	
Total release (ppm)				0.599		0.442				0.012				3.858		0.276				0.009	
		*Analysis of variance for nickel in subgroup 3*										*Analysis of variance for nickel in subgroup 4*									
*Source of variance*		*Sum of squares*		*Degree of freedom (DF)*		*Mean square*		*Variance ratio (F)*		*p-value*		*Sum of squares*		*Degree of (DF) freedom*		*Mean square*		*Varian ratio(F)*		*p-value*	
Between materials		0.037		2		0.018		2.713		0.107		1.848		2		0.924		5.319		0.022	
Within days		0.082		12		0.007						2.084		12		0.174					
Total		0.119		14								3.932		14							

In present study, release of nickel and chromium from space maintainers was measured on 1, 7, 14, 21 and 28 days keeping each subgroup in 100 ml of artificial saliva.

*Inductively coupled plasma*: Optical emission spectro-photometer was used to analyze nickel and chromium from space maintainers.

Total release of nickel in groups 1, 2, 3 and 4 at the given days was nil in groups 1 and 2 for Dantauram and Dtech, and 0.070 and 0.085 respectively, from Rocky mountain. In subgroup 3, it was 0.599, 0.442 and 0.012 for Dantauram, Rocky mountain and Dtech respectively. In subgroup 4, Dantauram showed maximum release of nickel out of three bands with a release of up to 3.858 ppm. For Rocky mountain and Dtech, the total release was 0.276 and 0.009 ppm respectively (Tables 1 and 2). As observed in our study as the number of space maintainers increased the release of nickel also increased. Similar, results were seen in a study done by Bhaskar V and Subba Reddy (2010)^1^ which also showed the increase proportionality of number of space maintainers with nickel release.

Total release of chromium in subgroups 1 and 2 was nil for Dantauram, Dtech and Rocky Mountain except for the 1st day in Rocky mountain group release on day 1 was 0.008 ppm. In subgroup 3, the total release of chromium from 1 to 28 days for Dantauram, Rocky mountain and Dtech was 0.002,0.684 and 0.012 ppm respectively, and in subgroup 4, it was 0.018,0.068 and 0.009 ppm for the three groups repectively (Tables 3 and 4). It was observed that with an increasing time frame the release of chromium decreased and similar results were seen in a study done by Bhaskar V and Subba Reddy (2010).^1^

In the present study, it was observed that, as the immersion time increased in all subgroups, there was a decrease in nickel and chromium release from all space maintainers. Nickel and chromium release was nil in all subgroups on 28th day. Similarly in a study done by Subba Reddy (2010),^1^ they also observed that release of nickel and chromium was minimum on 28th day of immersion.

In present study, the total release of nickel in artificial saliva ranged between 0.020 and 1.524 ppm (Tables 1 and 2) and the total release of chromium in artificial saliva ranged between 0.020 and 0.289 ppm (Tables 3 and 4). These values obtained in the present study were below the average daily dietary intake of these two metals. The dietary intake of nickel ranges from 300 to 500 ppm per day while chromium intake varies from 50 to 200 ppm per day.^5^

Although in present study, artificial saliva was used as an immersion/solution media to produce a realistic picture of human saliva to determine the release of nickel and chromium from different space maintainers, however, it would not produce natural oral environment for degradation of space maintainers for nickel and chromium.

A direct comparison between the values obtained in the present *in vitro* study cannot be compared with *in vivo* studies because of variation in methodology. In the oral cavity various factors, such as temperature, quantity and quality of saliva, plaque, physical and chemical properties of food and liquids and oral health conditions may also influence the results. In addition, composition of saliva may be affected by many physiological variables, such as time of the day, health conditions, diet and salivary flow rate may also affect the outcome.

The results of the present study suggests that there was no substantial release of nickel and chromium from space maintainers made of Dantaurum, Rocky mountain and Dtech which could cause any toxicity.

Further, studies are needed to determine the amount of metal ions released from space maintainers and orthodontic stainless steel appliances.

**Table 3:** **Table 3:** Release of chromium ions in subgroups 1 and 2 in ppm

				*Release of chromium ions in subgroup 1 in ppm*									*Release of chromium ions in subgroup 2 in ppm*								
*Days*				*Dantaurum*		*Rocky mountain*				*Dtech*				*Dantaurum*		*Rocky mountain*				*Dtech*	
1				Nill		Nill				Nill				Nill		0.008				Nil	
7				Nill		Nill				Nil				Nill		Nill				Nil	
14				Nill		Nill				Nil				Nill		Nill				Nil	
21				Nill		Nill				Nil				Nill		Nill				Nil	
28				Nil		Nil				Nil				Nil		Nil				Nil	
Total release (ppm)				Nill		Nill				Nill				Nill		0.008				Nill	
		*Analysis of variance for nickel in subgroup 1*										*Analysis of variance for nickel in subgroup 2*									
*Source of variance*		*Sum of squares*		*Degree of freedom (DF)*		*Mean square*		*Variance ratio (F)*		*p-value*		*Sum of squares*		*Degree of (DF) freedom*		*Mean square*		*Varian ratio(F)*		*p-value*	
Between materials		0.000		2		0.000		-		-		0.001		2		0.000		1.000		0.397 NS	
Within days		0.000		12		0.000						0.005		12		0.000					
Total		0.000		14								0.006		14							

NS: Nonsignificant

**Table 4:** **Table 4:** Release of chromium ions in subgroups 3 and 4 in ppm

				*Release of chromium ions in subgroup 3 in ppm*									*Release of chromium ions in subgroup 4 in ppm*								
*Days*				*Dantaurum*		*Rocky mountain*				*Dtech*				*Dantaurum*		*Rocky mountain*				*Dtech*	
1				Nill		0.142				0.012				Nill		0.037				Nil	
7				0.002		0.253				Nil				0.015		0.031				Nil	
14				Nill		0.289				Nil				0.003		Nill				0.009	
21				Nill		Nill				Nil				Nill		Nill				Nil	
28				Nil		Nil				Nil				Nil		Nil				Nil	
Total release (ppm)				0.002		0.684				0.012				0.018		0.068				0.009	
		*Analysis of variance for nickel in subgroup 3*										*Analysis of variance for nickel in subgroup 4*									
*Source of variance*		*Sum of squares*		*Degree of freedom (DF)*		*Mean square*		*Variance ratio (F)*		*p-value*		*Sum of squares*		*Degree of (DF) freedom*		*Mean square*		*Varian ratio(F)*		*p-value*	
Between materials		0.061		2		0.031		4.940		0.027		0.000		2		0.000		1.479		0.267 NS	
Within days		0.074		12		0.006						0.002		12		0.000					
Total		0.135		14								0.002		14							

NS: Nonsignificant

## CONCLUSION

Total release of nickel and chromium from all band and loop space maintainers ranged from 0.020 to 1.524 ppm and 0.002 ppm to 0.289 ppm respectively. The release of nickel and chromium was very much below when compared with the average dietary intake of nickel (300-500 ppm/day) and for chromium which ranges from (50-200 ppm/day) which were not capable of causing any toxic effects. There was no significant difference of release between the space maintainers made with different band materials.
